# Newly isolated terpenoids (covering 2019–2024) from *Aspergillus* species and their potential for the discovery of novel antimicrobials

**DOI:** 10.1007/s13659-025-00501-2

**Published:** 2025-03-18

**Authors:** Olusesan Ojo, Idris Njanje, Dele Abdissa, Tarryn Swart, Roxanne L. Higgitt, Rosemary A. Dorrington

**Affiliations:** 1 Department of Biochemistry, Microbiology and Bioinformatics, Rhodes University, Makhanda, South Africa; 2 Department of Chemical Sciences, Lead City University, P.O. Box 30678, Ibadan, Oyo State Nigeria; 3 Department of Chemistry, College of Natural Sciences, Jimma University, P.O Box 378, Jimma, Ethiopia

**Keywords:** Antimicrobial resistance, ESKAPE pathogens, Fungi, Secondary metabolites, Anti-infective activities

## Abstract

The rapid emergence of drug-resistant microbial pathogens has posed challenges to global health in the twenty-first century. This development has significantly made most antibiotics ineffective in the treatment of infections they cause, resulting in increasing treatment costs and annual death rates. To address the challenge posed by these pathogens, we explore the potential of secondary metabolites from *Aspergillus* species as a source of new and effective therapeutic agents to treat drug-resistant infections. Terpenoids, a distinct group of natural products, are extensively distributed in plants and fungi, and have been attributed with significant antibacterial, anticancer, and antiviral activities. In this review, we present an overview of *Aspergillus* species, and review the novel terpenoids isolated from them from 2019 to April 2024, highlighting anti-infective activity against members of the ESKAPE pathogens. We further focus on the strategies through which the structural framework of these new terpenoids could be modified and/or optimized to feed a pipeline of new lead compounds targeting microbial pathogens. Overall, this review provides insight into the therapeutic applications of terpenoids sourced from *Aspergillus* species and the potential for the discovery of new compounds from these fungi to combat antimicrobial resistance.

**Graphical Abstract:**

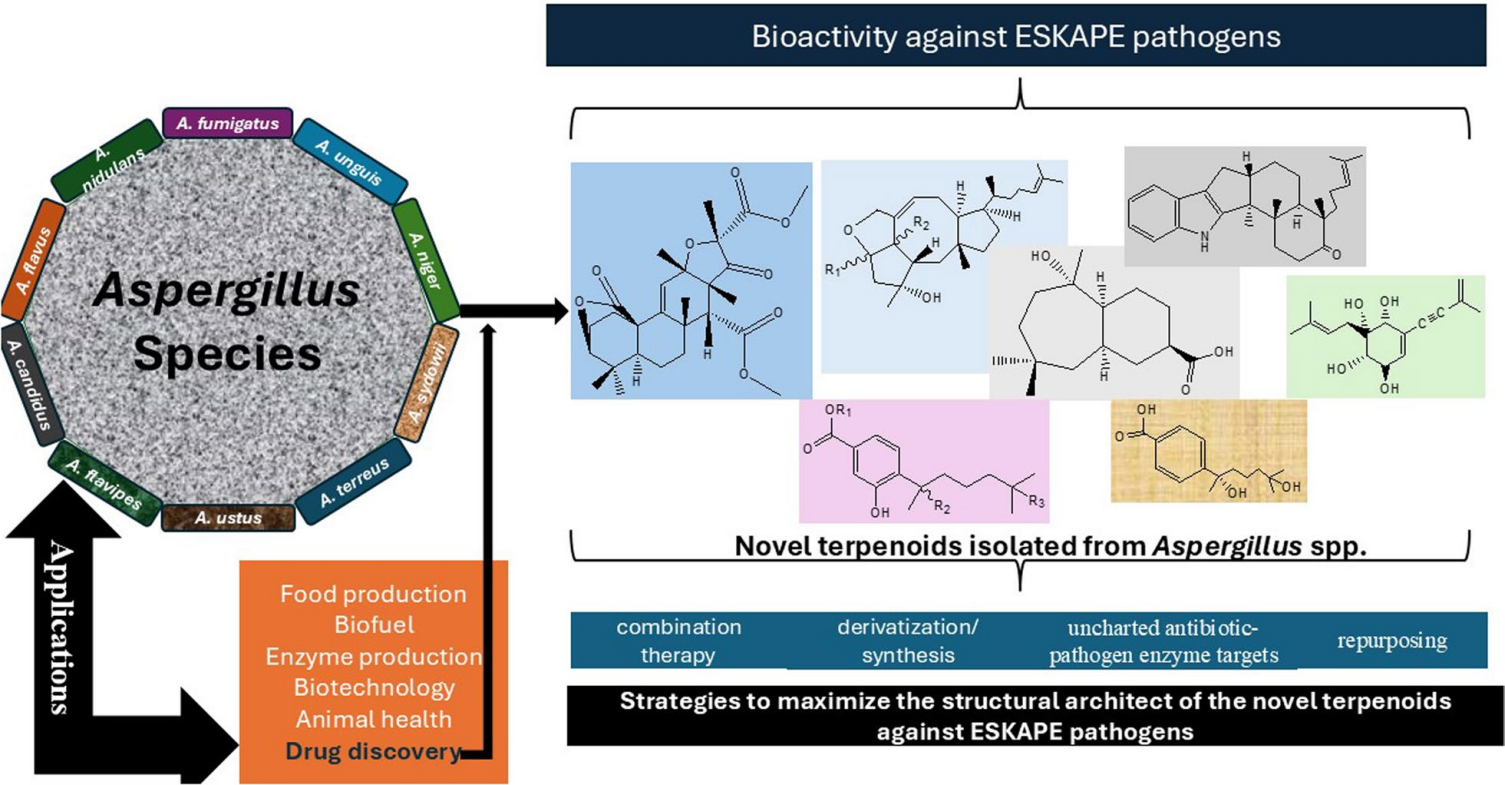

## Introduction

Antimicrobial resistance (AMR) unequivocally remains a major threat to global health in the twenty-first century. AMR occurs when microbes, such as bacteria, fungi, viruses, and parasites acquire resistance to one or more drugs [1]. Although AMR is an evolutionary process that happens with time as a result of alteration in the genetic materials of microorganisms [2, 3], the human “action and inaction” in the excessive and inappropriate use of antibiotics has immensely contributed to the evolution and continuous emergency of AMR [4]. Antimicrobial resistance has been noticed to almost all the clinical drugs that have been developed [3, 5, 6]. It has been estimated that AMR will be responsible for the death of 10 million people by 2050, and worse still, it will economically cost more than US$ 100 trillion annually if drastic and coordinated actions are not taken to curb it [7]. Among the microorganisms that have been implicated in antimicrobial resistance, ESKAPE pathogens (***E****nterococcus faecium*, ***S****taphylococcus aureus*, ***K****lebsiella pneumoniae*, ***A****cinetobacter baumannii*, ***P****seudomonas aeruginosa*, and ***E****nterobacter* species) are popular owing to their built-in ability to resist a large class of antibiotics [1, 8, 9]. The World Health Organization (WHO), in its first ever documentation on antibiotic-resistant "priority pathogens", identified and accredited them as the priority pathogens in the fight against AMR [10]. Controlling the AMR of these bacteria is predicated on developing and designing new antimicrobial drugs and/or alternative strategies.

From the time immemorial, nature has been regarded as a reservoir of bioactive molecules. Natural products (NPs) are biological molecules with low molecular weights, and can be obtained from marine or terrestrial plants, animals, and microorganisms [11]. Natural products (or secondary metabolites) are an unprecedented source of chemical diversity, with unique pharmacological properties [11, 12]. Natural products and derivatives have historically been acting as an alternative, invaluable source of therapeutic agents towards several ailments [13, 14]. More than 50% of clinically approved antibiotics currently on the market are sourced from nature [13]. It is noteworthy that the first blockbuster antibiotic, penicillin, isolated by Sir Alexander Fleming from a green fungal mold *Penicillium notatum* in 1928 changed the course of history, and saved millions of lives during World War II [15].

In nature, fungi are widely distributed and recognized as an emerging source of anti-infective agents, with unparallel and unmatched modes of actions [16, 17]. The genus *Aspergillus* is one of the largest genera in the fungal kingdom [18]. *Aspergillus* species has become a research hotspot recently among the scientific community due to their potent ability to biosynthesize unusual, rich, and matchless bioactive molecules [19–22]. Moreover, the past five years of research on the secondary metabolites from the *Aspergillus* has ushered in several new compounds, including peptides [23], alkaloids [24], and terpenoids [25]. These new natural products have been linked with several pharmacological actions, including activities against ESKAPE pathogen members [26–29]. In the absence of comprehensive, holistic, and standalone review on *Aspergillus*-derived terpenoids in the past five years [30–33], the central purpose of this work is to review and document newly reported terpenoids isolated from the *Aspergillus* in different habitats (covering 2019–April 2024), and examine their pharmacological actions against ESKAPE pathogens. We also delve into different ways these novel frameworks of terpenoids could be maximally or optimally utilized. Overall, the current work would afford us to gain further insight into the therapeutic roles of these terpenoids in the fight against AMR and open new research paradigm in future studies.

## Methodology

### Search strategy and inclusion criteria

The databases of Google Scholar, ScienceDirect, Springer, Scopus, and PubMed were utilized in the search for literature in this review using single or combination of key words—“*Aspergillus*”, “*Aspergillus* AND natural products”, *Aspergillus* secondary metabolites”, *Aspergillus* AND terpenoids”, *Aspergillus* natural products AND ESKAPE pathogens, “*Aspergillus* species AND secondary metabolites”, and “*Aspergillus* AND bioactive compounds”. The search covered publications from January 2019–April 2024. The search was refined to include only research studies that reported new terpenoids from the *Aspergillus* and/or their pharmacological actions against any ESKAPE pathogen. Only articles published in English language were included in the data search. Data was thoroughly screened, and relevant papers (those reported newly isolated terpenoids) were collated using Zotero reference manager.

### Exclusion criteria

Abstracts, conference proceedings, commentaries, editorial and case reports that did not meet the inclusion criteria were excluded. Similarly, new terpenoids isolated from the co-culture of *Aspergillus* species with other organisms were excluded as shown in a previous study [34]. This is to document the new terpenoids specifically derived from *Aspergillus* species. Besides, we noticed some co-culture experiments lack controls during our data collection to ascertain whether the metabolites were really produced by the *Aspergillus* species.

## The ESKAPE pathogens: to what extent have they “escaped”?

The ESKAPE pathogens account for the majority of resistant nosocomial infections worldwide [35, 36] and the incidence of resistance in these pathogens is ever-increasing. The acronym “ESKAPE” was coined by Louis B. Rice in 2008 [35] to highlight ***E****nterococcus faecium*, ***S****taphylococcus aureus*, ***K****lebsiella pneumoniae*, ***A****cinetobacter baumannii*, ***P****seudomonas aeruginosa*, and ***E****nterobacter* species based on their ability to “escape” the pharmacological actions of most current antibiotics [35]. Non-susceptible strains can be resistant to (i) at least one antimicrobial drug in three or more antimicrobial categories (multiple drug resistance; MDR), (ii) all antimicrobial agents except in two or less antimicrobial categories (extensive drug resistance; XDR), or (iii) all antimicrobial agents in all antimicrobial categories (pandrug resistance; PDR) [36]. The core resistance mechanisms involve modification of antibiotic targets, antibiotic influx prevention, biochemical modification of the antibiotics, and overexpression of efflux pumps, and have been comprehensively reviewed elsewhere [37–39]. The increase in drug-resistance in ESKAPE pathogens has resulted in diverse variants. Among them are carbapenem-resistant *K. pneumoniae* (CRKP) [40, 41], carbapenem-resistant *A. baumanii* (CRAB) [40], carbapenem-resistant *P. aeruginosa* (CRPA) [41], carbapenem-resistant *Enterobacteriaceae* (CRE), and multi-drug-resistant *A. baumannii* [42]. Others are vancomycin-resistant *Enterococci* (VRE), vancomycin-intermediate *S. aureus* (VISA) [43], vancomycin-resistant *E. faecium* (VRE), vancomycin-resistant *S. aureus* (VRSA), methicillin-resistant *S. aureus* (MRSA), and fluoroquinolone-resistant *P. aeruginosa* (FRPA) [44] to mention a few.

## *Aspergillus* species: an overview of a genus of ‘friends’, or ‘foes’ with benefits

*Aspergillus* is a genus of spore-forming mold first described in 1729 by an Italian botanist, Pier Antonio Micheli. This genus includes more than 200 identified species that are characterized by the morphology of a conidophore (a long-chained projection of conidia or spores) [45]. *Aspergillus* is widely distributed across the globe and species are mainly found in the soil as saprophytes, where they survive on dead organic matter and play an important role in the recycling of elemental carbon and nitrogen through the environment. However, some species live in water, air and on vegetation [45].

The genus comprises species of positive and negative economic importance in medicine, agriculture, and industry. Anxiously, the world is medically sensing a threat currently posed by *Aspergillus* infections. About 12% of the species are known as major causes of life-threatening infections, including aspergilloma, invasive aspergillosis, and otomycosis in humans. The most representative culprits are *A. flavus*, *A. terreus*, *A. felis, A. fumigatus*, *A. niger*, and *A. nidulans* [45]. It is estimated that more than 300, 000 people develop aspergillosis yearly, with about 30 million people at risk [46]. The agricultural sector is not left out from the negative impact of *Aspergillus* species. They threaten global food security and safety initiatives. For instance, *Aspergillus* species contaminates agricultural commodities, such as cereal grains with toxins (aflatoxins and ochratoxins), leading to economic loss and food shortage [47]. It is more worrisome that consumption of aflatoxin-contaminated foods has great health implications. There is risk of developing cancers, especially liver cancers in human due to mycotoxin exposure, although no statistically significant link/association has been documented in the literature [48].

**Fig. 1:**
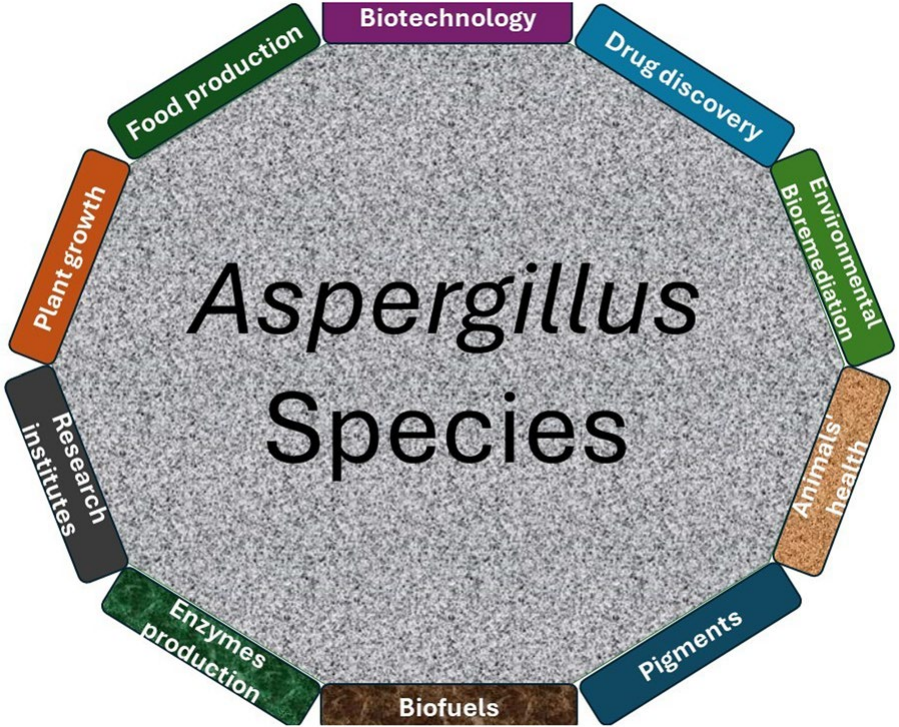
An overview of some of the applications of the *Aspergillus* in medicine, agriculture, biotechnology, ecosystem, and industries

Regardless of the pathogenic role of *Aspergillus* species in our ecosystem, the genus has shaped biotechnology, food, and medical industries over the past 100 years (Fig. 1). The culprit *A. terreus*, for instance, has been utilized industrially in bio-based production of itaconic acid and the drug lovastatin. *A. niger* has gained industrial application in fermentation-based production of proteins and enzymes. These species, in addition to other culprits, are indeed human ‘enemies’ with enormous benefits [49]. Today, the impact of *Aspergillus* species in drug discovery has been much pronounced with respect to the number of bioactive secondary metabolites, including terpenoids isolated from the genus.

## Terpenoids

### Basic chemical classifications of terpenoids and their biosynthesis

Terpenoids form a specialized group of organic compounds biosynthesized by most plants, animals, and microorganisms, including bacteria and fungi. There are over 90, 000 known terpenoids with diverse biological activities. The name “terpene” was first used by August Kekulé in 1863 to describe a chemical compound, turpentine/terpentine, derived from tree plant *Pistacia terebinthus* [50]. All terpenoids are formed biosynthetically from isopentenyl pyrophosphate (IPP) and its structurally distinct isomer, dimethylallyl pyrophosphate (DMAPP), via either a mevalonic acid pathway (MVA) or a 2-C-methylerythritol phosphate (MEP) (also called, non-mevalonate) pathway [51]. While MVA pathway is common in eukaryotes and the archaea, MEP is utilized by higher plants, eubacteria, and algae. Here, we summarize the MVA pathway as it is commonly utilized by most organisms.

**Fig. 2:**
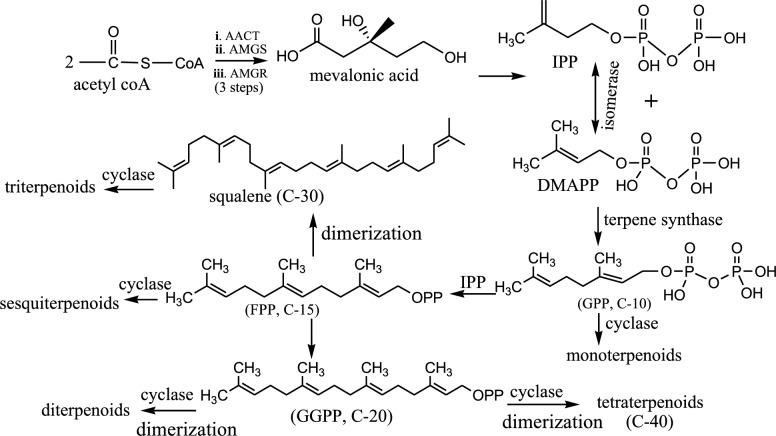
Biosynthetic pathway of terpenoids via mevalonic acid [51]. *AACT* acetoactl-CoA thiolase, *HMGS* hydroxymethylglutaryl-CoA synthase, *HMGR* hydroxymethylglutaryl-CoA reductase, *OPP* diphosphate moieties, *IPP* isopentenyl pyrophosphate, *DMAPP* dimethylallyl pyrophosphate, *GPP* geranyl diphosphate, *FPP* farnesyl diphosphate, *GGPP* geranyl–geranyl-diphosphate

Firstly, IPP and its isomer DMAPP are joined in a “head-to-tail” manner under the influence of an enzyme (terpene synthase) to initiate the formation of geranyl diphosphate (GPP, C-10) chain. When GPP combines with a unit of IPP, farnesyl diphosphate (FPP, C-15) is produced. Then, FPP initiates the formation of geranyl–geranyl- diphosphate (GGPP, C-20) when joined with another unit of IPP. The biosynthesized GGPP can enzymatically dimerize to produce C-40 chain length, from where carotenoids (tetraterpenoids) are formed. Likewise, enzymatic dimerization of FPP (C-15) can initiate the formation of squalene (C-30), a precursor of triterpenoids. Under the influence of enzyme cyclases, these varying chain lengths of carbon atoms cyclize to form different ring sizes and numbers of various terpenoids. Other biochemical transformations, including oxidation, substitution, de-substitution and rearrangement reactions can occur, leading to diverse terpenoid natural products (Fig. 2). Based on the isoprene principle, C-5 molecule, as proposed by Croatian-Swiss scientist, Leopold Ružicka [52], terpenoids could be distinctively classified into several groups, namely hemi-terpenoids (C-5), monoterpenoids (C-10), sesquiterpenoids (C-15), diterpenoids (C-20), triterpenoids (C-30), and tetraterpenoids (C-40).

### Quantification of terpenoids isolated from the *Aspergillus* species

**Fig. 3:**
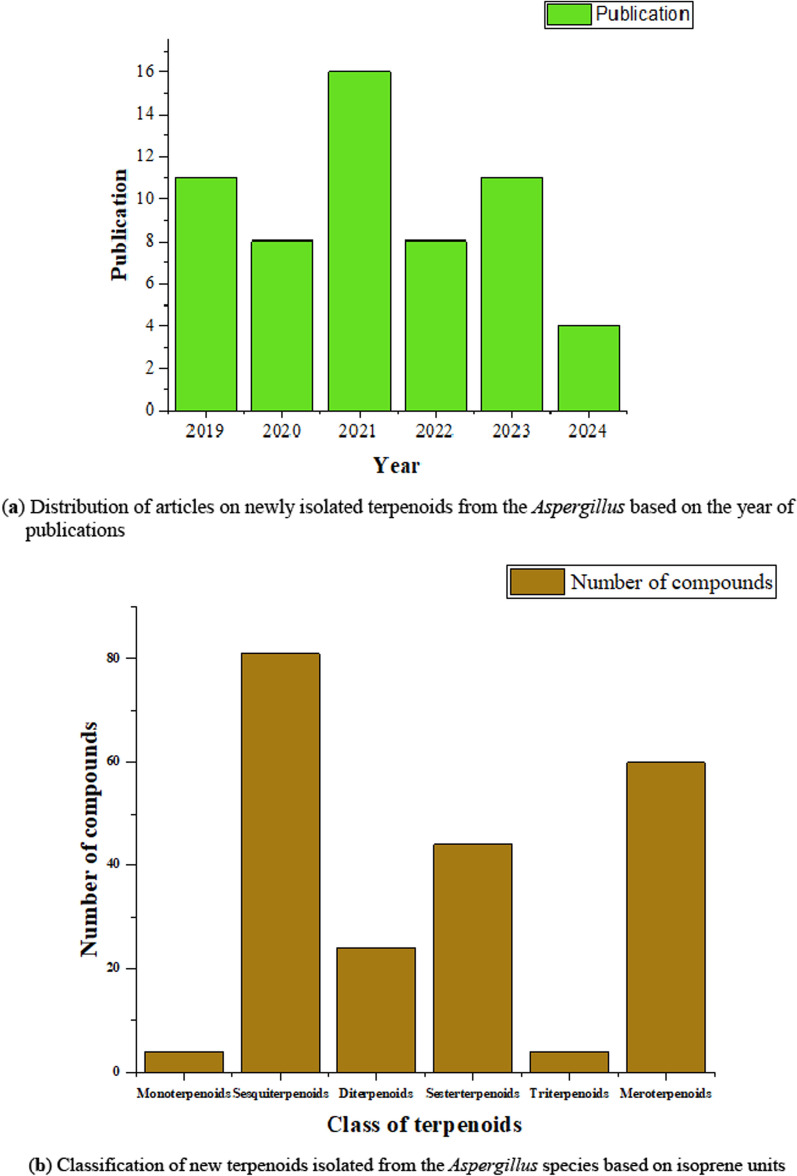

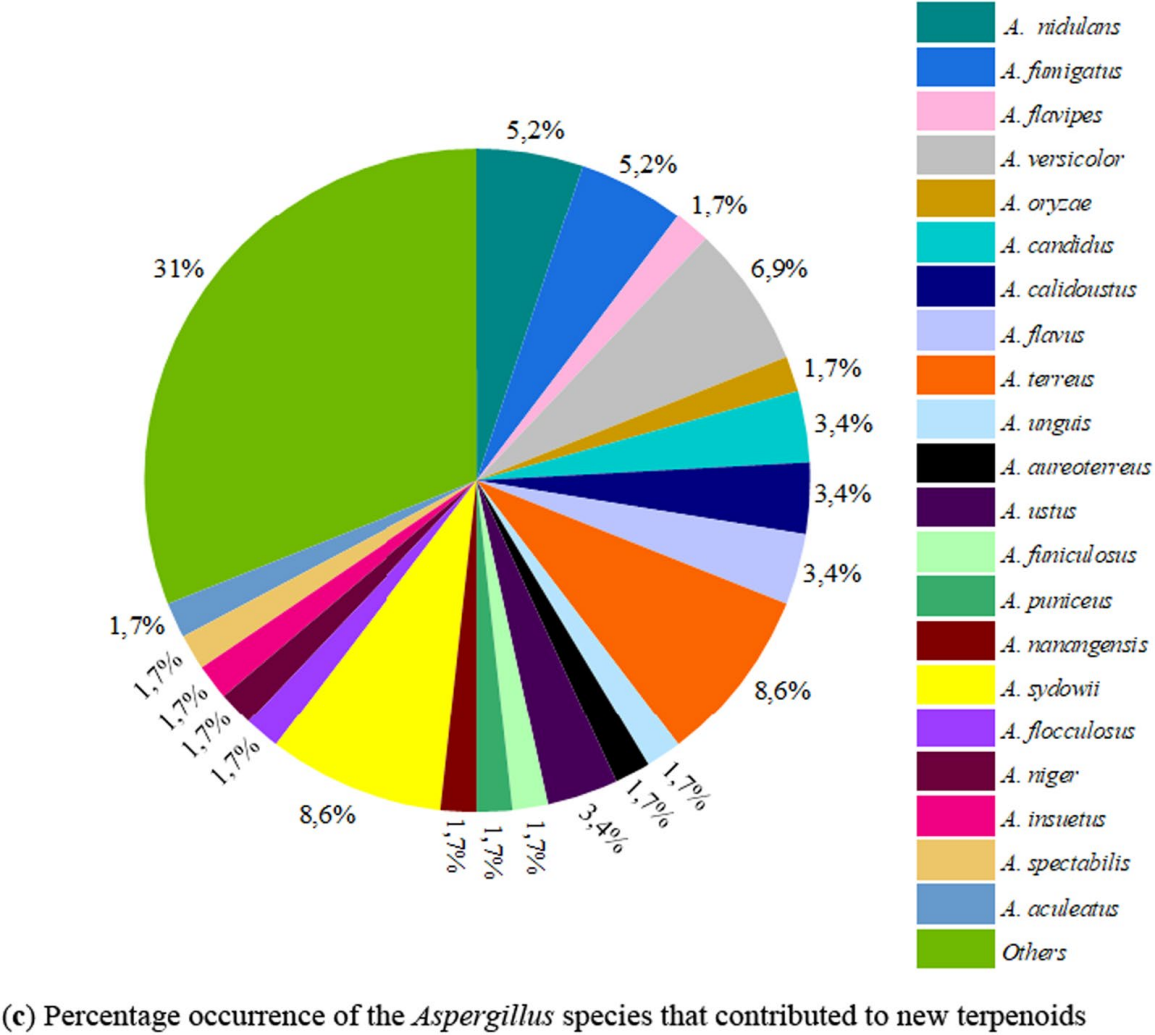
** a–c** Quantification of new terpenoids and the *Aspergillus* species that produced them

After extensive search and careful screening of the published articles from 2019 to April 2024 using the named databases, a total of 58 articles met our selection criteria for newly isolated terpenoids from the *Aspergillus* species. As shown in Fig. 3a, majority of the articles were published in 2021, followed by 2019 and 2023. During these studied periods, a total of 217 new terpenoids were isolated from the *Aspergillus* species and unambiguously characterized using spectroscopic methods. The most newly isolated terpenoids belong to the class sesquiterpenoids, followed by the meroterpenoids, and only a few monoterpenoids and triterpenoids were reported (Fig. 3b). More than 22 species of *Aspergillus* contributed to these new terpenoids. Notably, *A. terreus* (8.62%) and *A. sydowii* (8.62%) are the most studied *Aspergillus* species as shown by the number of occurrences, followed by *A. versocolor* (6.90%). However, most members of the genus (denoted as ‘*Others*’) are not fully characterized taxonomically at the species level as shown in Fig. 3c. Hence, full identification of these *Aspergillus* ‘species’ (denoted as ‘*Others*’) offers many research possibilities from the taxonomic perspective (Fig. 3c).

## Chemical diversity of terpenoids isolated from the *Aspergillus* species

**Fig. 4:**
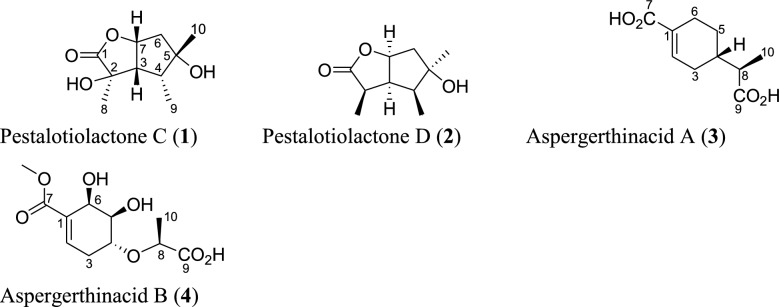
Structures of monoterpenoids isolated from *Aspergillus* species

**Fig. 5:**
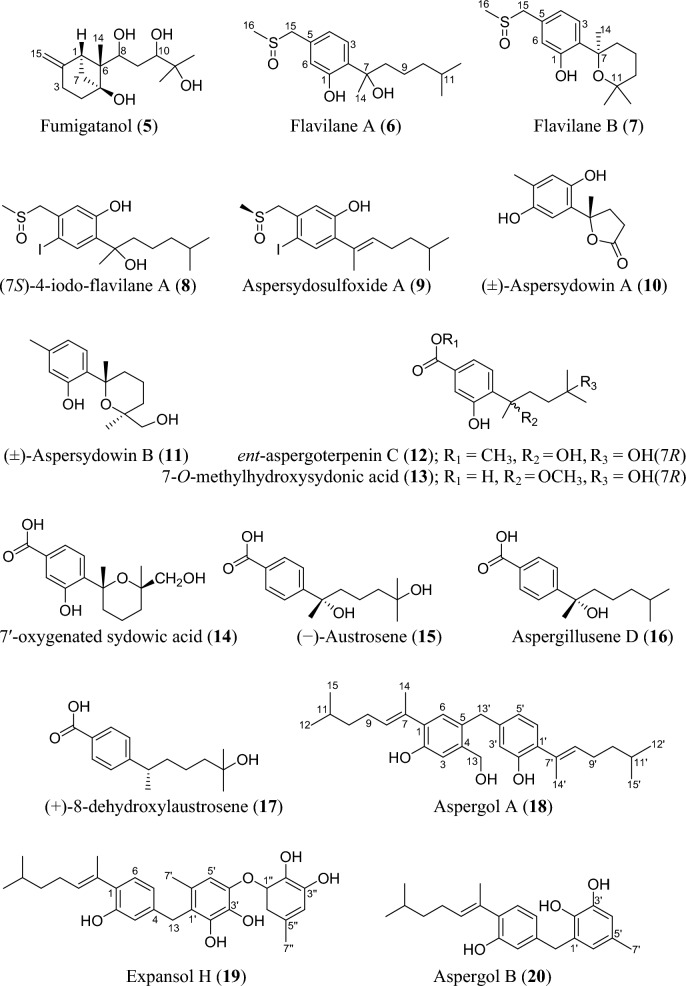

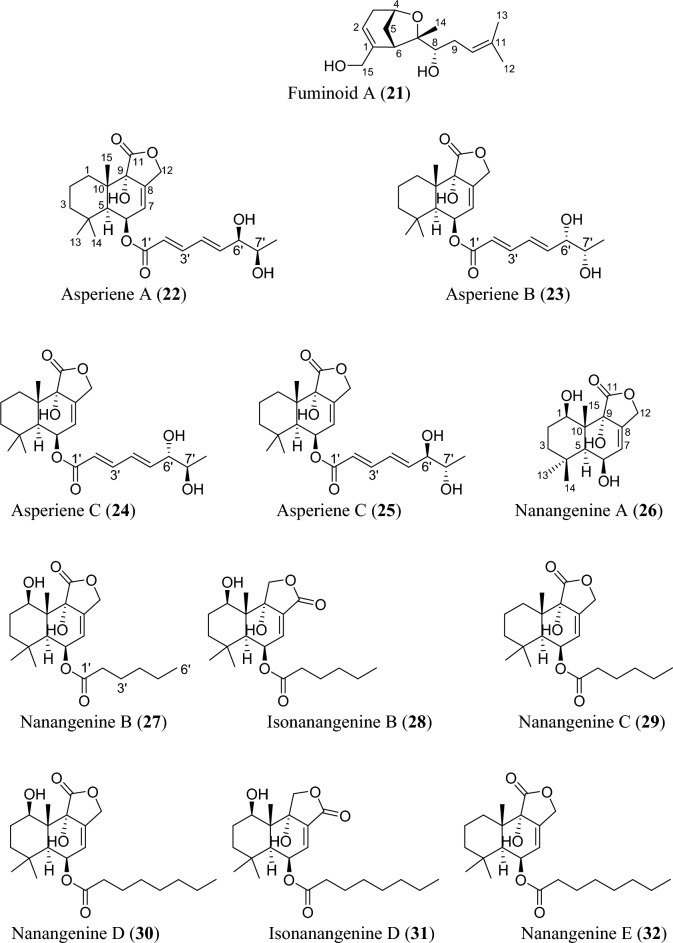

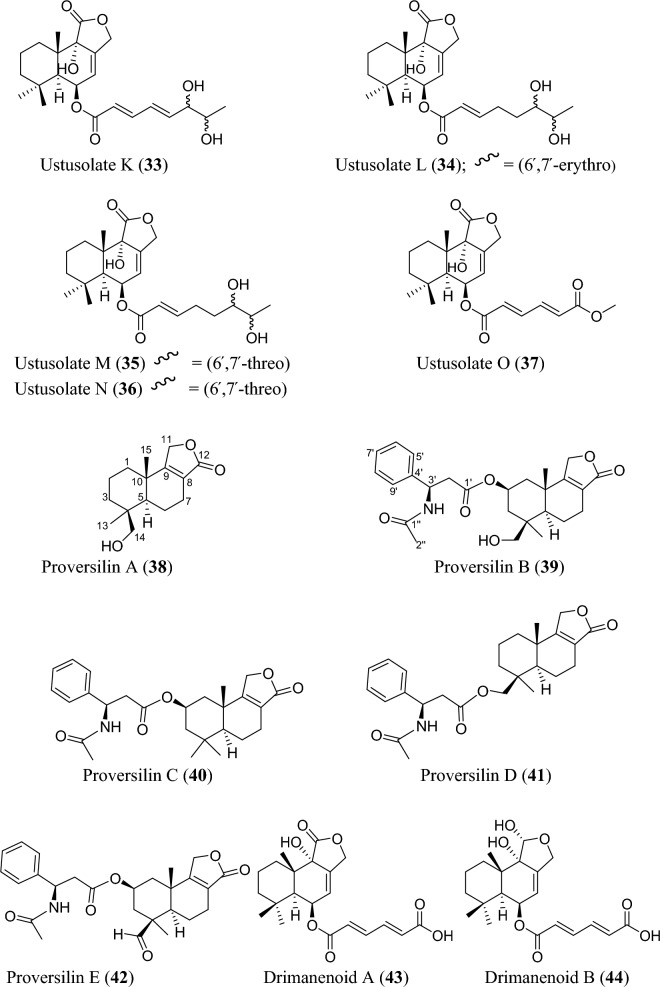

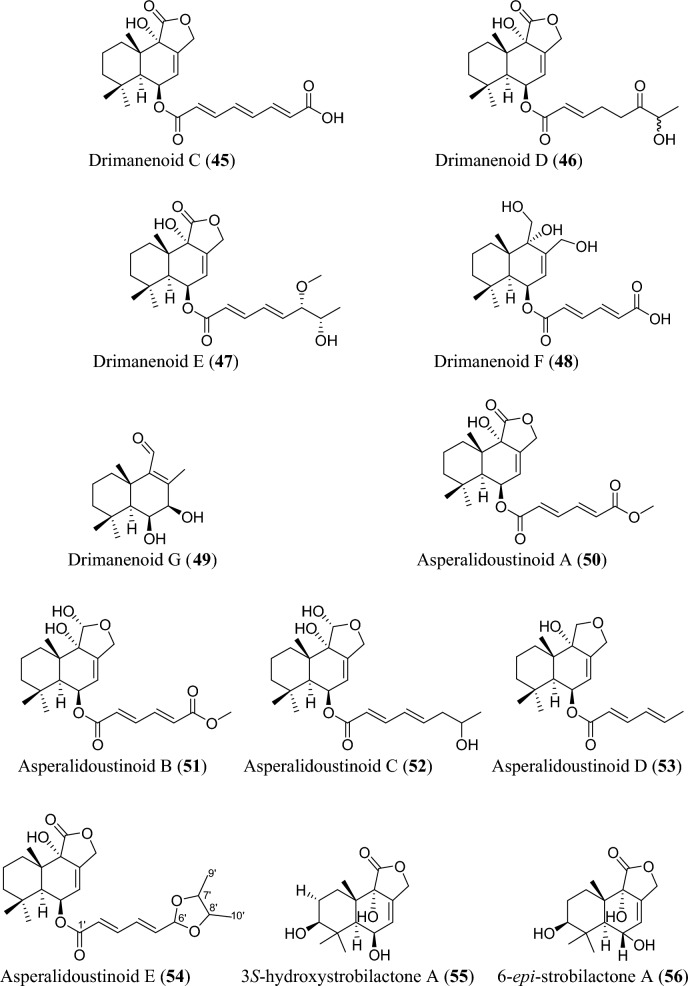

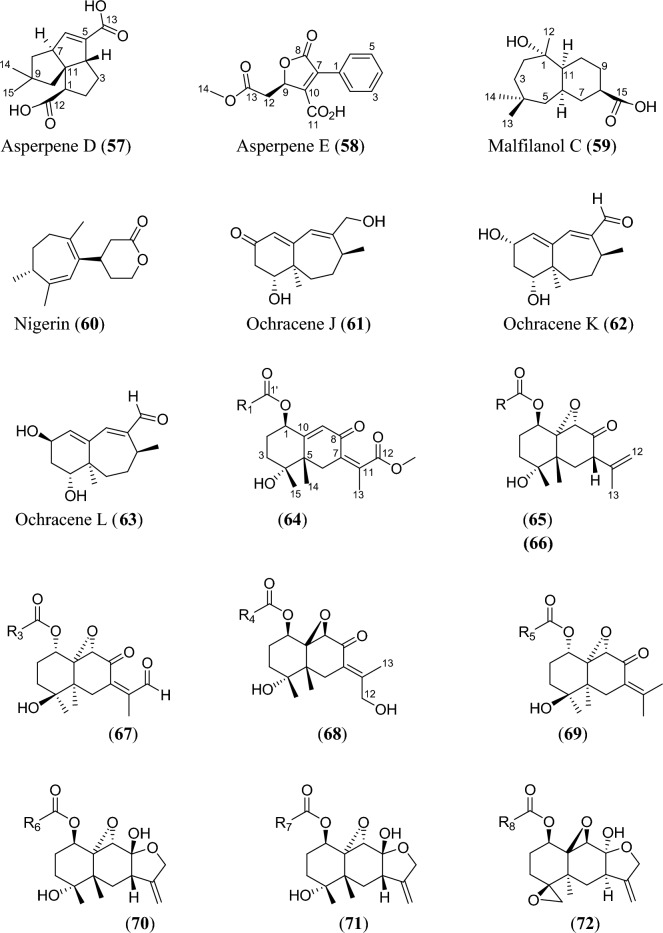

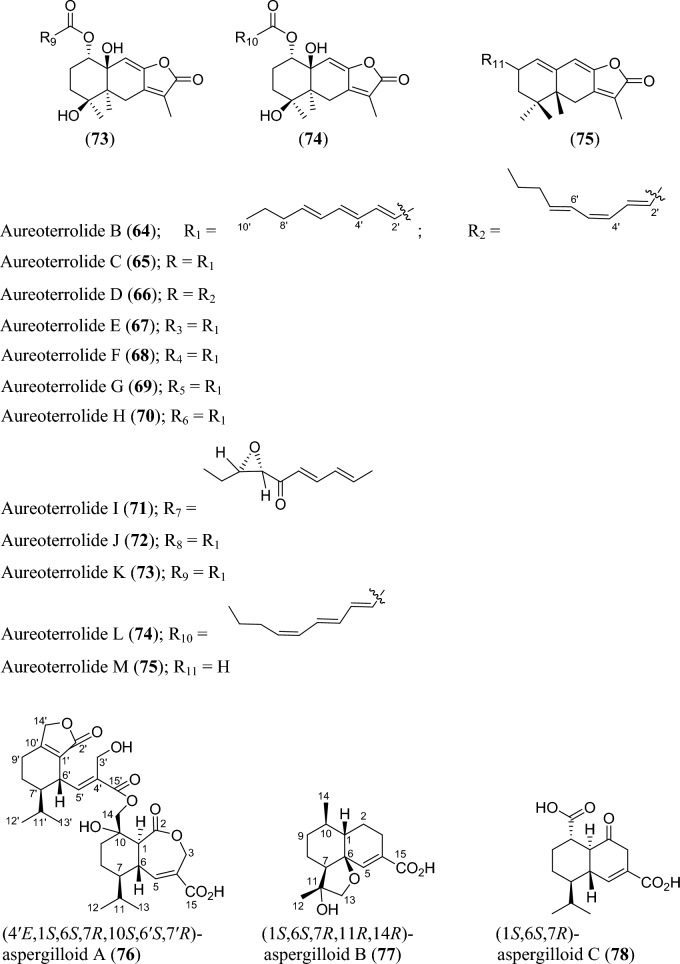

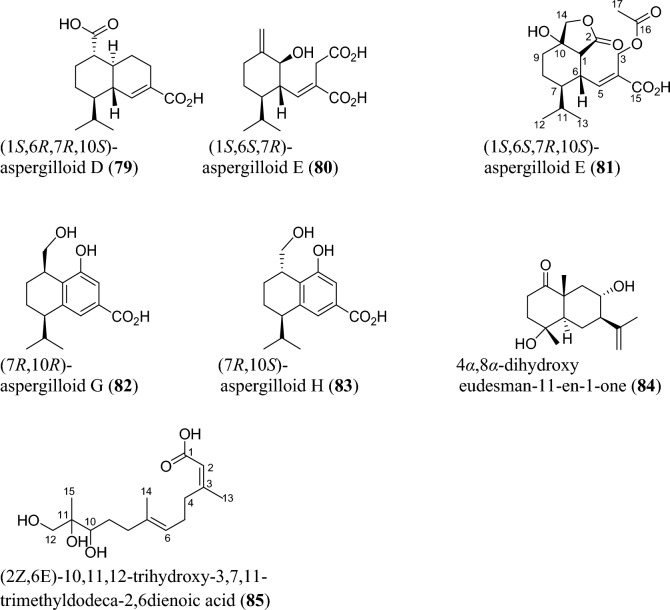
Structures of sesquiterpenoids isolated from *Aspergillus* species

**Fig. 6:**
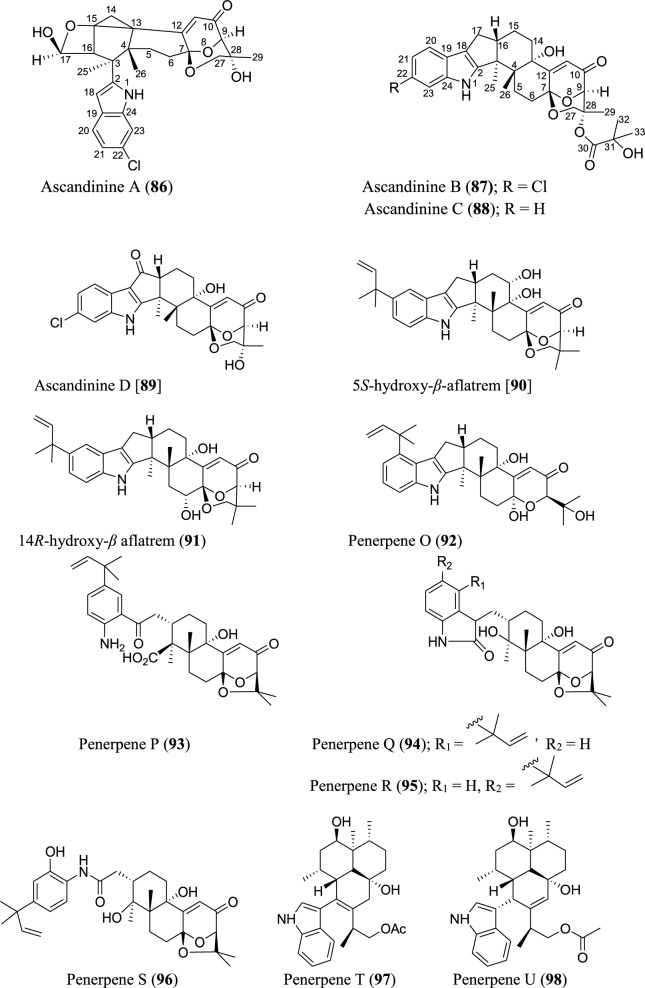

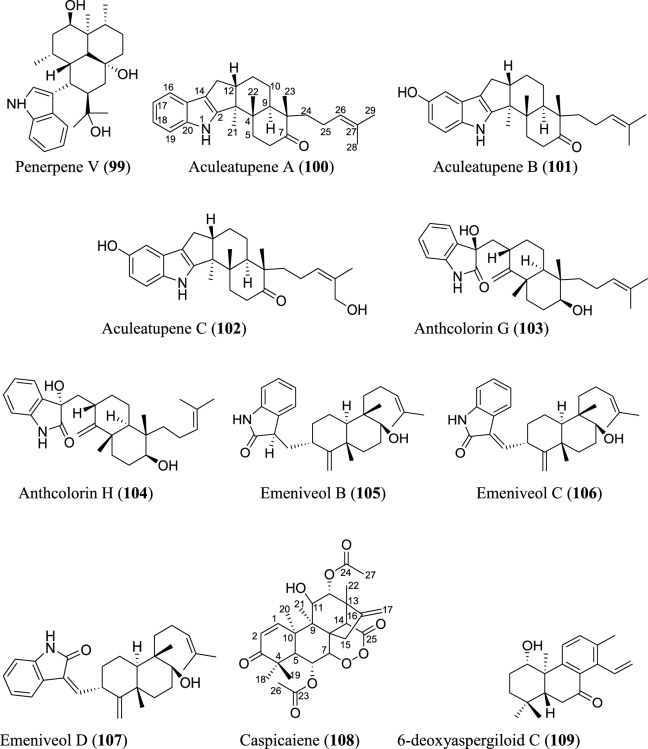
Structures of diterpenoids isolated from *Aspergillus* species

**Fig. 7:**
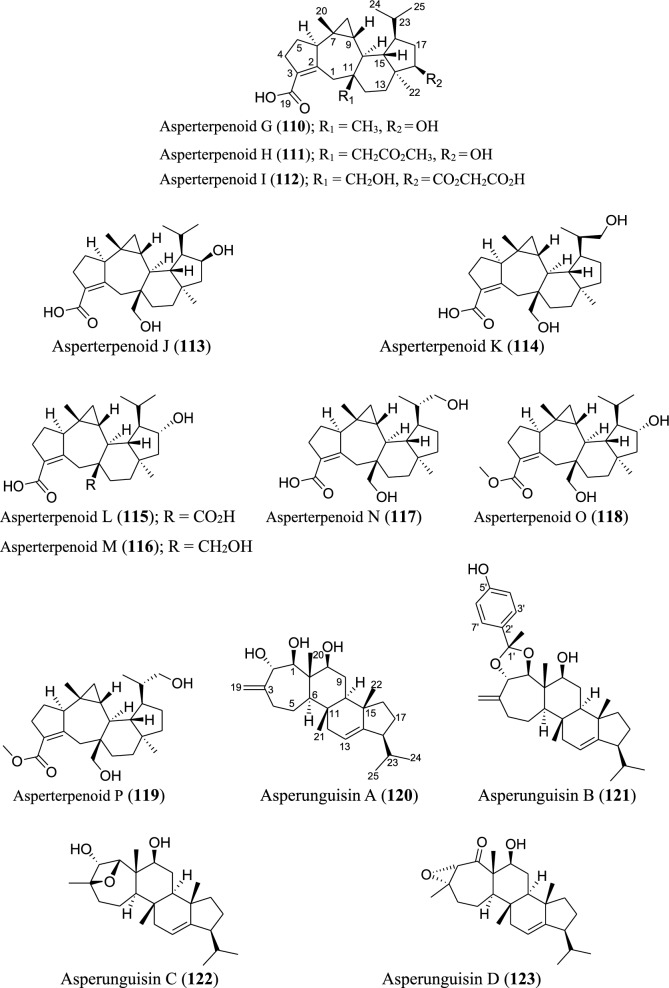

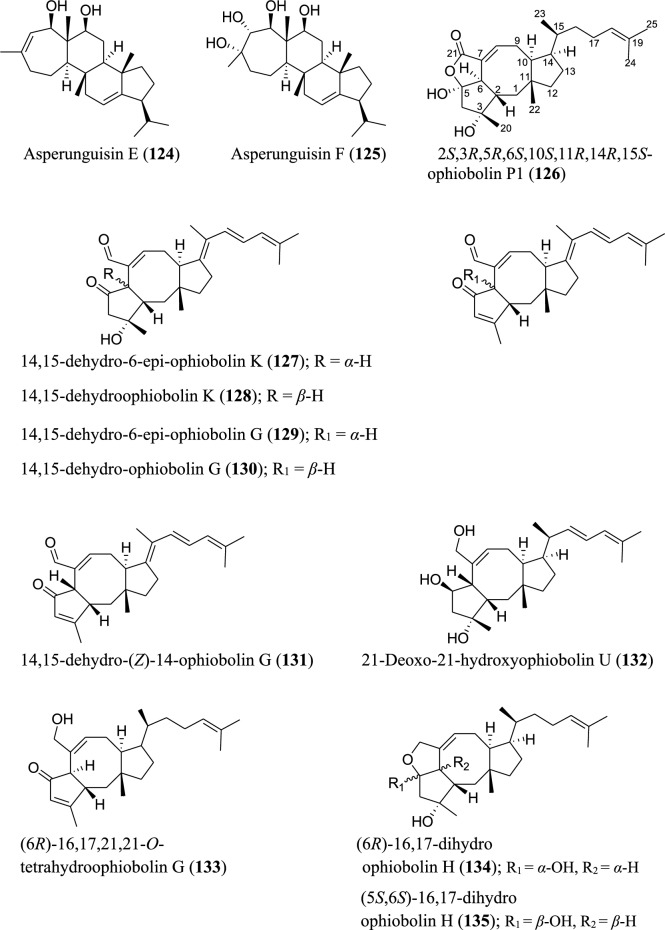

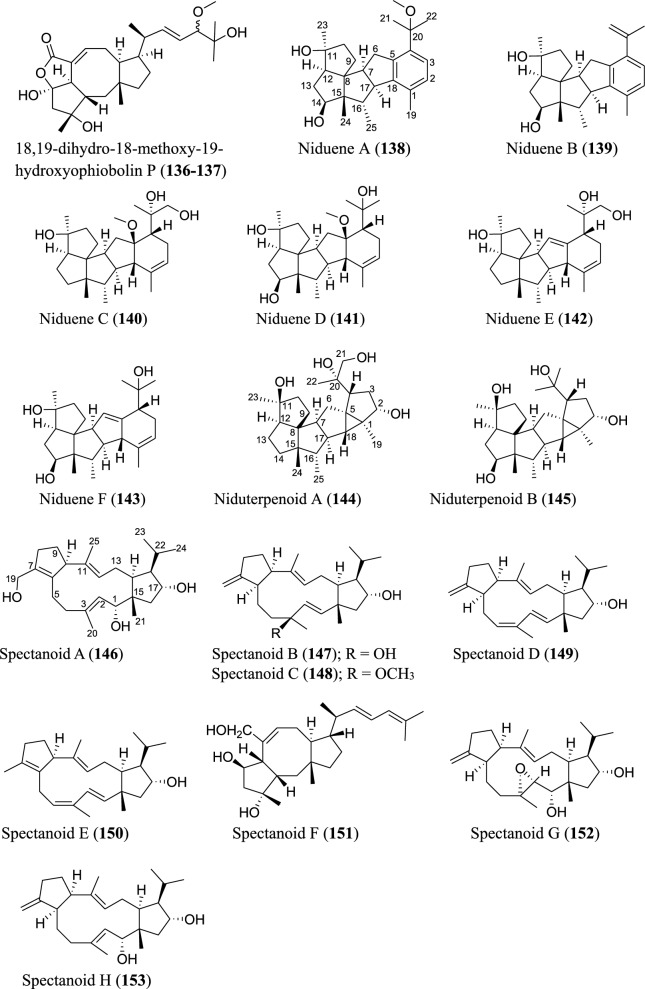
Structures of sesterterpenoids isolated from *Aspergillus* species

**Fig. 8:**
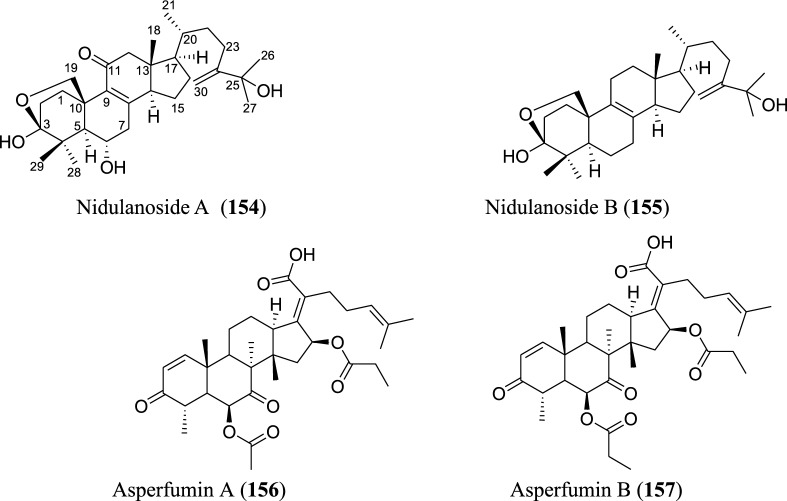
Structures of triterpenoids isolated from *Aspergillus* species

**Fig. 9:**
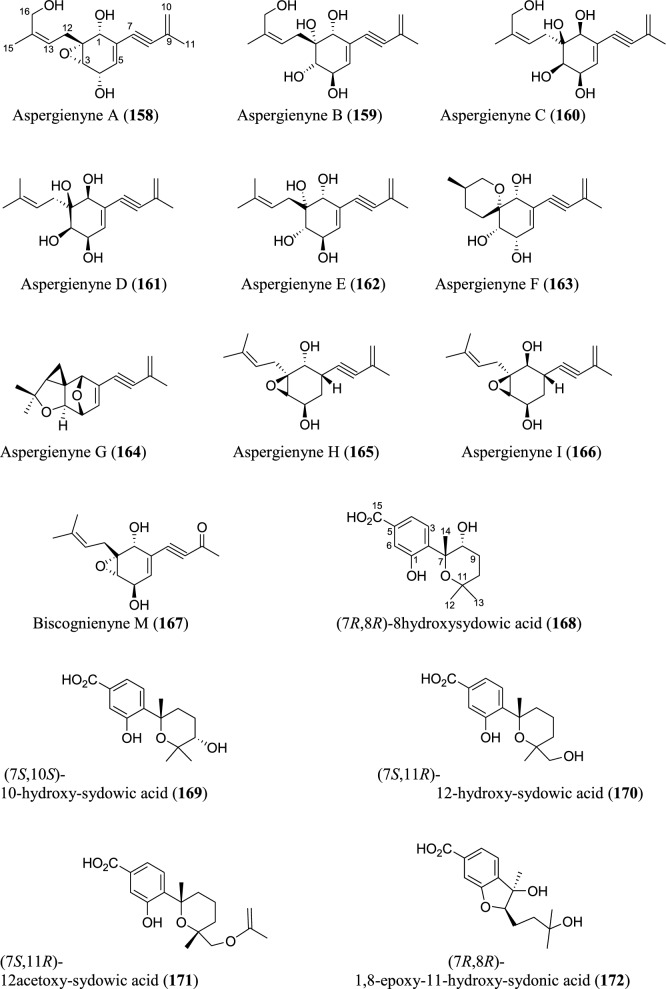

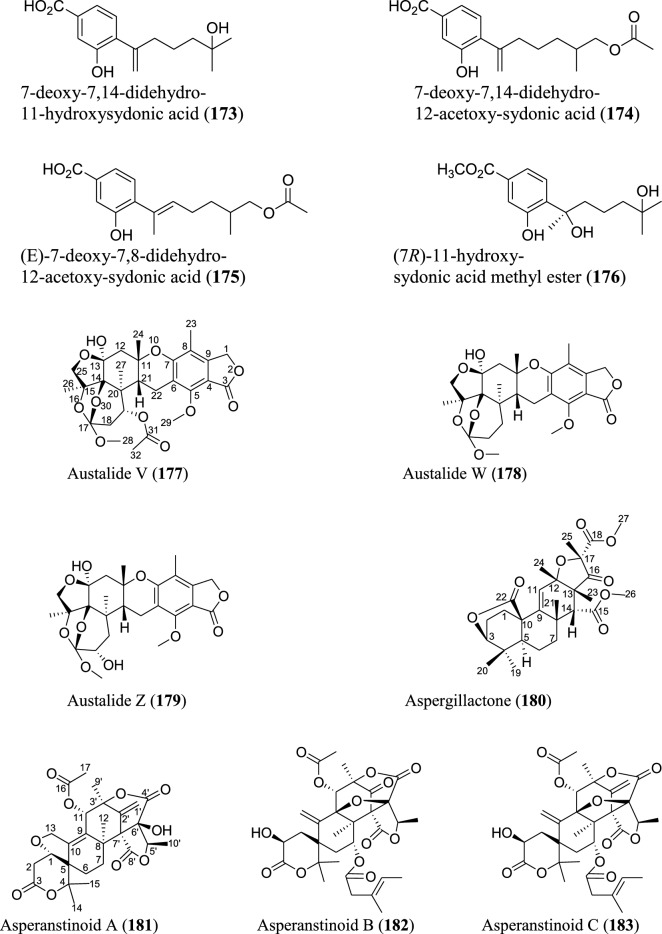

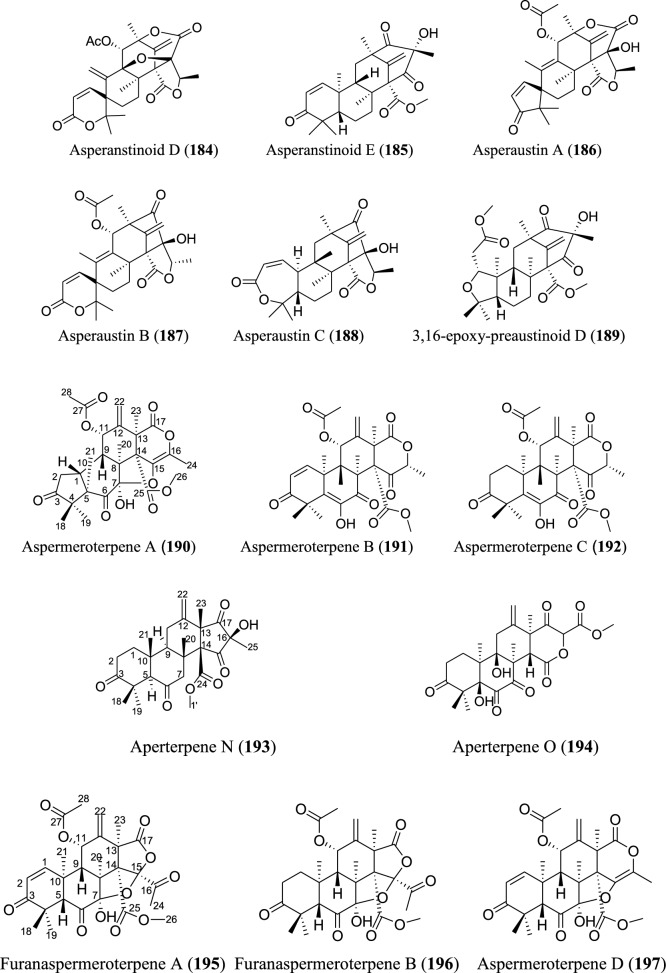

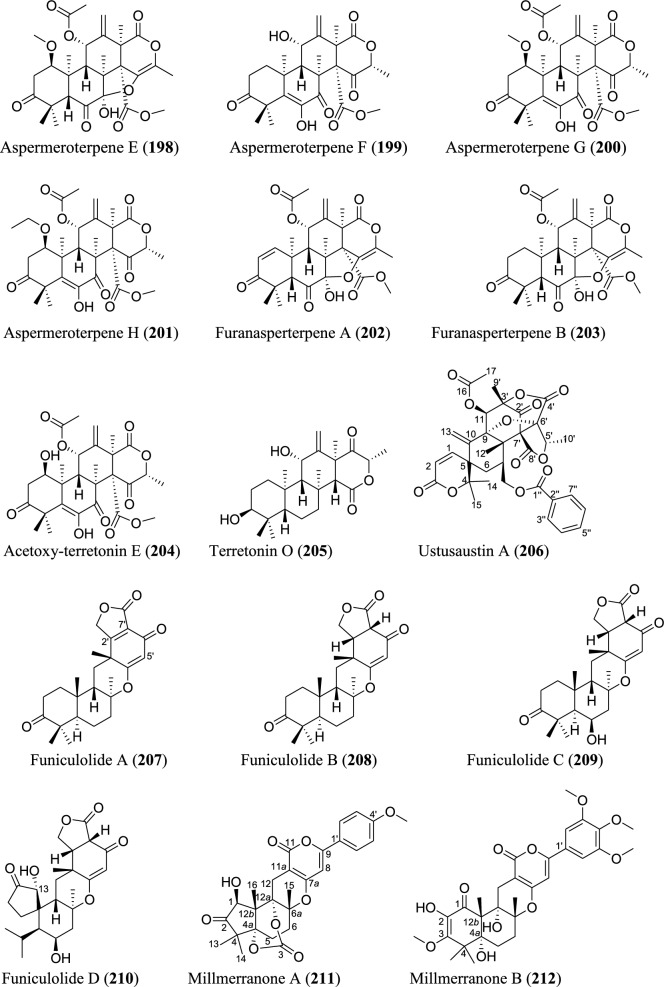

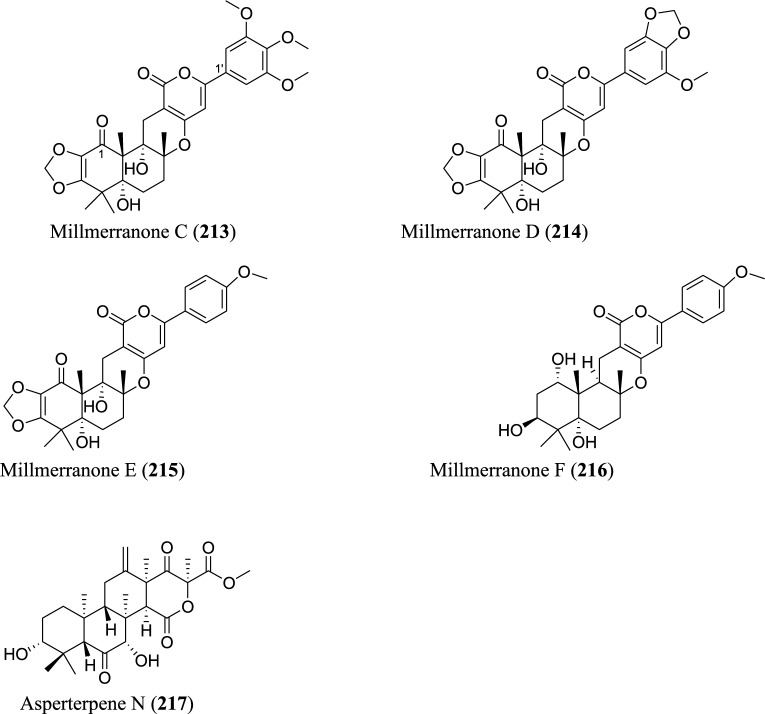
Structures of meroterpenoids isolated from *Aspergillus* species

Several novel frameworks of terpenoids (Figs. 4, 5, 6, 7, 8, 9) have been isolated from the *Aspergillus* species. In this section, the novel molecules identified in each of the classes of terpenoids, including meroterpenoids are discussed.

### Monoterpenoids

Only four monoterpenoids were isolated from the *Aspergillus* species in the past five years as shown in Fig. 4. These include pestalotiolactones C-D (**1–2**) which are two new butyrolactone-skeleton monoterpenoids isolated from deep-sea fungus *A. versicolor* SD-330 [53]. Aspergerthinacids A and B (**3–4**) are the other two new monoterpenes identified from the fungal strain *Aspergillus* sp. CYH26 isolated from the Chinese plant *Cynanchum bungei* Decne [54].

### Sesquiterpenoids

Sesquiterpenoids are abundant in the genus *Aspergillus* and 81 compounds were isolated. They belong to different sesquiterpenoid skeletons, including bergamotane-type (**5**), bisabolane-type (**6–21**), drimane-type (**22–56**), humulane-derived (**61–63**), eremophilane-type (**64–75**), cadinene-type (**76–83**), and eudesmane-type sesquiterpenoid (**84**) among others (Fig. 5).

To start with, one previously undescribed compound, fumigatanol (**5**), was recently reported from the fungus *A. fumigatus* M1 isolated from the plant *Aconitum brevicalcaratum* Diels. [55]. In a study published recently, a pair of new enantiomers ( ±)-flavilane A (**6**) together with a new derivative flavilane B (**7**) was identified from marine-derived fungus, *A. flavipes* 297 isolated from the fresh seawater in China. Compounds (**6**) and (**7**) are rare cases of bisabolane-type sesquiterpenoids with methylsulfinyl moiety [56]. In addition to these flavilanes, an iodo-derivative of flavilane A was recently reported from cold-seep fungus *A. sydowii* 10–31 collected in Taiwan Island. The compound (7*S*)-4-iodo-flavilane A (**8**) adds to the sesquiterpenoid molecular diversity, bearing both sulphur and iodine [57]. Similarly, a new sulphur-containing bisabolane sesquiterpenoid has been identified. The compound, aspersydosulfoxide A (**9**), was isolated from the marine-derived *A. sydowii* LW09 [58]. ( ±)-aspersydowin A (**10**) and ( ±)-aspersydowin B (**11**) were isolated from the fungus *A. sydowii*. The two compounds are enantiomers [59]. Likewise, *ent*-aspergoterpenin C and 7-O-methylhydroxysydonic acid (**12–13**) were isolated from deep-sea sediment-derived fungus *A. versicolor* SD-330 collected in South China Sea [53]. Compounds 7′‑oxygenated sydowic acid and ( −)-austrosene (**14–15**) were discovered from the fermented culture of *Aspergillus* sp. SCSIO06786 obtained from the deep-sea sediment [60]. Meanwhile, after much scrutiny, compound (**14**) was reported a year after as a new isolated bisabolene sesquiterpenoid obtained from *A. versicolor* SD-330 [61]. Aspergillusene D (**16**) was discovered from the fermented culture of sponge-derived fungus *A. sydowii* SCSIO 41301 harvested from the Xisha Islands in China [62]. ( +)-8-dehydroxylaustrosene (**17**), a new bisabolane sesquiterpenoid, was reportedly isolated and characterized from marine fungus *A. sydowii* BTBU20213012 [63]. Aspergol A (**18**), expansol H (**19**) and aspergol B (**20**) featuring bisabolene carbon framework were isolated from marine-derived *Aspergillus* sp. MCCC 3A00392 collected at central Pacific Ocean [64]. Fuminoid A (**21**)**,** a new compound with a bicyclo[3.2.1]octane ring isolated from the *Huperzia serrata*-derived fungus *A. fumigatus*, represents an unusual bisabolane scaffold via the transformation of the methyl [65].

In addition to these sesquiterpenoids, four drimane sesquiterpene esters were isolated from the strain *A. flavus* derived from the marine environment. Notably, the asperienes A–D (**22–25**) are epimers, and differed only in the side chain configuration at C-6′, and C-7′. Structurally, compounds **22** and **23** possess *erythro* configuration (6′*R*,7′*R* or 6′*S*,7′*S*) at C-6′ and C-7′ while compounds **24** and **25** have *threo* configuration (6′*S*,7′*R* or 6′*R*,7′*S*) at C-6′ and C-7′ [66]. Similarly, seven new drimane sesquiterpenoids (**26–32**) have been reported from the fermented culture of a novel Australian fungus *A. nanangensis*. Structurally, nanangenine A (**26**) features as trihydroxylated drimane lactone. While nanangenines B-E (**27,29–30, 32**) bear C6′/C8′ acyl side chains, isonanangenines B and D (**28, 31**) bear isomeric lactone rings [67]. Ustusolates K–O (**33–37**) represent five sesquiterpenoids with drimane skeleton recently isolated from *Aspergillus* sp. RR-YLW-12 derived from marine organism, *Rhodomela confervoides*. Ustusolate K features 6′,7′-diol in the side chains in addition to two double bonds at C2′ and C4′. However, ustusolates M and N (**35–36**) were obtained as inseparable epimers [68]. Proversilins A−E (**38–42**) form new sets of drimane-type sesquiterpenoids discovered from *A. versicolor* F210 isolated from the plant *Lycoris radiata* bulbs. Structurally, proversilins B-E distinctively possess *N*-acetyl-*β*-phenylalanine moiety, representing first examples of natural products with such moiety [69]. Drimanenoids A−G (**43–49**) were recently discovered from *Aspergillus* sp. NF2396 ethyl acetate extracted derived from an earwig. These compounds structurally feature an unsaturated fatty acid side chain at C-6′ with conjugated double bond ranging from 1–3, and a terminal carboxyl or methyl group [70]. Asperalidoustinoids A–E (**50–54**) were isolated from wetland soil-derived fungus *A. calidoustus*. Asperalidoustinoid E (**54**) distinctively features a rare dioxolane moiety [71]. Compounds (**55–56**), reported as new drimane sesquiterpenoids, were discovered from the fermented culture of algae-derived *Aspergillus* sp. RR-YLW-12 isolated from the strain *Rhodomela confervoides* [25].

Again, asperpenes D and E (**57–58**) were chromatographed and identified from the fungal strain *Aspergillus* sp. SCS-KFD66 ethyl acetate extract isolated from bivalve mollusk, *Sanguinolaria chinensis* [72]. Bicyclic malfilanol C (**59**) was identified from ethyl acetate extract of *A. puniceus* A2 derived from deep-sea sediment [73]. Chemical profiling of marine sponge-derived *A. niger* 164,117 collected in the South China Sea resulted in nigerin (**60**) and ochracenes J-L (**61–63**). While ochracenes J-L form new sets of humulane-derived sesquiterpenoids, the nigerin represents a new sesquiterpene scaffold, featuring an unprecedented 1-(3-*n*-pentyl)-2,5,6-trimethyl-cycloheptane ring system. [74]. Highly oxygenated twelve compounds, characterized as unusual eremophilane sesquiterpenes with an oxidized C-4 and named as aureoterrolides B-M (**64–75**), were obtained from the culture of the fungus *A. aureoterreus*. Isolate aureoterrolide J (**72**) represents first pentacyclic furanoeremophilane, comprising a 3/6/6/5/3 and a 3,6-spiro ring system [75]. Compounds (**76–83**) possessing cadinene-sesquiterpene skeleton were identified and elucidated from fungus *A. flavus* isolated from the toxic plant, *Tylophora ovata* [76]. Similarly, one novel eudesmane-sesquiterpene, 4α,8α-dihydroxyeudesman-11-en-1-one (**84**), has been reportedly isolated from endophytic fungus *A. flavus* [76]. Meanwhile, chemical investigation of ethyl acetate extract obtained from fungal strain *Aspergillus* sp. SCSIO 41029 isolated from deep sea gave only linear sesquiterpene (**85**) identified in these reviewing years [77].

### Diterpenoids

Diterpenoids form an integral part of the *Aspergillus* secondary metabolites as revealed in the data obtained. Statistically, a total of twenty-four diterpenoids were isolated. Most of the isolated diterpenoids possess an indole skeleton (Fig. 6). Ascandinines A−D (**86–89**) are new indole diterpenoids isolated from an Antarctic sponge-derived fungus *A. candidus* HDN15-152. Ascandinine A (**86**) possesses a 6/6/6/6/6 pentacyclic ring system with an indole substitute and a 2oxabicyclo[2.2.2]octan-3-ol motif, while ascandinines B−D (**87–89**) possess a rare carbon skeleton, featuring 6/5/5/6/6/6/6-fused ring system [78]. Heptacyclic 5*S*-hydroxy-*β*-aflatrem (**90**) and 14*R*-hydroxy-*β* aflatrem (**91**), paxilline-type indole diterpenes, were identified from *Sphagneticola trilobata*-derived fungus *Aspergillus* sp. PQJ-1 [79]. Similarly, eight new indole-diterpenoids, penerpenes O–V (**92–99**), were recently isolated from *Aspergillus* sp. ZF-104 derived from marine soft coral. Penerpenes R and S (**95–96**) possess an unusual indolin-2-one units in their structures [80]. Three indole diterpenoids were obtained from *A. aculeatus* KKU-CT2 derived from a contaminated laboratory agar plate. The new pentacyclic diterpenoids, aculeatupenes A–C (**100–102**), feature a 6/5/5/6/6 ring structure [81]. Anthcolorins G–H (**103–104**), oxoindolo-diterpene epimers, were discovered from a culture of the endophytic fungus *A. versicolor* isolated from mangrove *Avicennia marina* fruit [82]. Three new oxoindolo-type diterpenoids have been recently reported. The emeniveol B–D (**105–107**) were isolated from marine-derived *Aspergillus* sp. MCCC 3A00392 collected at the central Pacific Ocean. Notably, emeniveol C is the *cis*-isomer of emeniveol D [64]. Caspicaiene (**108**) possessing a kaurene diterpenoid nucleus with 6/6/6/5/6 ring system structure was reported from the culture of endophytic *Aspergillus* N830 isolated from the plant *Gleditsia caspia* desf. [83]. A cleistanthane diterpenoid, 6-deoxyaspergiloid C (**109**), was isolated from the *Aspergillus candidus* [84].

### Sesterterpenoids

Several new sesterterpenoids have been identified from *Aspergillus* species. Specifically, forty-four new metabolites were recorded as depicted in Fig. 7. For instance, ten new sesterterpenoids were isolated from *A. oryzae*. The asperterpenoids G-P (**110–119**) feature pentacyclic skeleton with an unusual 5/7/3/6/5 ring pattern [85]. Asperunguisins A−F (**120–125**), asperane class of sesterterpenoids, were identified from a culture of endolichenic fungus *A. unguis* isolated from the lichen *Xanthoria* sp. These asperunguisins were uniquely characterised by hydroxylated 7/6/6/5 tetracyclic system [86]. Ophiobolin P1 (**126**) was recently reported from a wetland soil-derived fungus *A. calidoustus* [71]. Similarly, five previously undescribed ophiobolin-group sesterterpenoids (**127–131**) have been reported from the marine fungal strain *A. flocculosus* isolated from the seaweed *Padina* sp. [87]. 21-Deoxo-21-hydroxyophiobolin U (**132**) was recently reported from fungus *Aspergillus* sp. RR-YLW-12 derived from marine red algae, *Rhodomela confervoides* [68]. In addition to these ophiobolins, three new compounds (**133–135**) belonging to this type of sesterterpenoids were identified from a deep sea-derived fungus *A. insuetus* SD-512 extract obtained from cold seep sediments [88]. Compound (**136**) and its isomer (**137**) were newly ophiobolin sesterterpenoids discovered from the fermented culture of algae-derived *Aspergillus* sp. RR-YLW-12 isolated from the strain *Rhodomela confervoides*. Notably, compound (**137**) is a C18 epimer of 18,19-dihydro-18-methoxy-19-hydroxyophiobolin P (**136**) [25]. Six functionalized sesterterpenoids named niduenes A−F (**138–143**) were discovered from endophytic fungus *A. nidulans*. These new niduenes are characterized by an unprecedented 5/5/5/5/6 pentacarbocyclic ring system. Intriguingly, niduenes A and B (**138–139**) feature the first pentacarbocyclic sesterterpenoids with aromatic nucleus [89]. Likewise, two highly congested compounds (**144–145**) with hexacarbocyclic 5/5/5/5/3/5 ring system have been reported from solid cultivation of *A. nidulans*. The carbon skeleton of the two compounds structurally devoid of unsaturated functional groups. Notably, the two isolated compounds represent the first set of hexacyclic sesterterpenoids [90]. Spectanoids A–H (**146–153**) were isolated from the fungus *Aspergillus spectabilis* obtained from *Artemisia* grassland. Spectanoids A-G consist of tricarbocyclic skeleton, with a rare 5/12/5 ring arrangement [91].

### Triterpenoids

Triterpenoids, though few, are parts of the secondary metabolites isolated from *Aspergillus* species as revealed by the analyzed data. In total, four new triterpenoids were introduced to the chemical diversity of the genus. These include two new 30-norlanostane triterpenoids, nidulanosides A (**154**) and B (**155**), which were isolated from fungus *A. nidulan* ethyl acetate extract. These compounds represent the first naturally occurring 30-norlanostane triterpenoids with a C9 side chain attachment at C_-17_, and a hemi-acetal formed between C_-3_ and C_-19_ [92] (Fig. 8). The other two, asperfumins A and B (**156–157**), were obtained from a chemical study on *A. fumigatu*, an endophytic fungus isolated from *Cleidion brevipetiolatum* fresh root [93].

### Meroterpenoids

Meroterpenoids are specialized forms of terpenoids with exceptionally diverse structures, ranging from simple to complex ones. Unlike their ‘normal’ terpenoid counterparts, they are characterized by mixed biosynthetic pathways, and broadly grouped as polyketide-terpenoids or nonpolyketide-terpenoids pathways [30]. Meroterpenoids are well distributed in *Aspergillus* (Fig. 9). They include aspergienynes A–I (**158–166**), diisoprenyl-cyclohexene class of meroterpenoids, that were recently isolated from mangrove-derived endophytic fungus *Aspergillus* sp. GXNU-Y65 [94]. Biscognienyne M (**167**), a new member of diisoprenyl-cyclohexene class of meroterpenoids, was isolated from the mangrove endophytic fungus *Aspergillus* QG1a [95]. Nine novel meroterpenoids (**168–176**) were discovered from solid rice culture of the endophytic fungus *A. versicolor* isolated from mangrove *Avicennia marina* fruit [82].

Austalide meroterpenoids V and W (**177–178**) were derived from *A. ustus* VKM F-4692 isolated from the building stone. Interestingly, these two compounds are the first sets of austalides with a 5/6/6/6/6/5/5 heptacyclic ring system [96]. Recently, austalide Z (**179**) was reported from soft coral-derived ethyl acetate extract of *Aspergillus* sp. [97]. A new 3,5-dimethylorsellinic acid (DMOA)-based meroterpenoid, aspergillactone (**180**), was reportedly identified from extracts of *Aspergillus* sp. CSYZ-1 [98]. Similarly, five (DMOA)-based meroterpenoids, trivially named as asperanstinoids A–E (**181–185**), were recently reported from soil-derived fungus *A. calidoustus*. Asperanstinoid A (**181**) exemplifies second form of (DMOA)-based meroterpenoid in nature, featuring an unusual 6/5/6/6/6/5-fused hexacycles and an uncommon “1,13-epoxy” moiety [99]. Asperaustins A−C (**186–188**) represent three new DMOA-derived meroterpenoids isolated from *Aspergillus* sp. fungus ZYH026 obtained from marine brown alga *Saccharina cichorioides*. Structurally, asperaustin A (**186**) with a unique 5/6/6/6/5 pentacarbocyclic skeleton features an unprecedented spiro[4.5]deca-3,6-dien-2-one attachment [100]. 3,16-epoxy-preaustinoid D (**189**), a new DMOA-based meroterpenoid, was isolated from wetland soil-derived fungus *A. calidoustus* [71]. Aspermeroterpene A–C (**190–192**) were obtained from the marine fungus *A. terreus* GZU31-1. Compound (**190**) represents first unprecedented and highly congested hexacyclic meroterpene with 5/3/6/6/6/5 carbon skeleton [101]. Aperterpenes N and O (**193–194**) were identified from the ethyl acetate extract of the fermented culture of endophytic fungal strain *A. terreus* EN-539 isolated from marine alga, *Laurencia okamurai* [102].

Two furanaspermeroterpenes A and B (**195–196**) were co-isolated in addition to five previously undescribed congeners aspermeroterpenes D–H (**197–201**) from fungal strain *A. terreus* GZU-31-1 derived from marine environment. While the two furanaspermeroterpenes A and B have unusual pentacyclic skeleton, and feature the first sets of DMOA-derived meroterpenoids in which five-membered D/E rings were coupled, aspermeroterpene D (**197**) possess a rare *cis*-fused A/B ring [103]. In addition to this, a study by the same author(s) had earlier reported the isolation of furanasperterpenes A and B (**202–203**) along with 11-acetoxy-terretonin E (**204**) from the same fungus strain *A. terreus* GZU-31-1. Structurally, the difference between these furanasperterpenes and the 11-acetoxy-terretonin E arises from furan ring formation between C_-7_ and C_-15_ in furanasperterpenes A and B [104]. Similarly, terretonin O (**205**) has been identified from methanolic extracts of marine *A. terreus* LGO13 and thermophilic *A. terreus* TM8 [105]. Ustusaustin A (**206**), a novel austin-type meroterpene, was isolated from the culture extract of *A. ustus* TK-5. Ustusaustin A features as the first example of 1′-nor-austin analogues with the possession of a rare 7-benzoylation [106]. Funiculolides A−D (**207–210**), four new fungal meroterpenoids, were reported from *A. funiculosus* CBS 116.56 by utilizing the heterologous expression of a cryptic gene cluster in the fungal strain. Intriguingly, these compounds unexpectedly and biosynthetically utilized 5-methylorsellinic acid (5-MOA) as against the DMOA [107]. Fermented culture of Australian fungus *Aspergillus* sp. CMB-MRF324 derived from pasture soil reportedly yielded six millmerranones A−F (**211–216**). Millmerranone A (**211**) distinguishably features a rare carbon ring bearing a unique cyclic carbonate, thereby making it an unusual meroterpenoid [108]. Asperterpene N (**217**) was discovered from the fermented culture of endophytic fungus *Aspergillus* sp. derived from *Tripterygium wilfordii*. Compound (**217**) is a unique DMOA-based meroterpenoid, featuring a *cis*-fused C/ D ring [109].

## Pharmacological activities against ESKAPE pathogens

A few pharmacological activities have been reported for some of the new terpenoids against at least one member of the ESKAPE pathogens. However, much work needs to be done to assay other compounds. Aspergillactone (**180**) was reported to display selective but effective antibacterial activity against four clinical strains of methicillin-resistant *S. aureus* (USA300, BKS231, BKS233 and ATCC 25923), with minimum inhibitory concentrations (MICs) of 2, 4, 8 and 16 μg/mL, respectively. However, it inhibited *A. baumannii*, *E. faecium*, *P. aeruginosa*, *E. faecalis*, and *K. pneumonia* at MICs > 32 μg/mL than the positive control [98]. Compound (**5**) inhibited the strain of *S. aureus* at a concentration of 256 μg/mL [55]. Flavilanes A and B (**6**–**7**) possess antimicrobial action against the human pathogen, *S. aureus*, with MICs of 64 and 32 μg/mL, respectively [56]. Compound (**14**) isolated from *A. versicolor* had inhibition against zoonotic human bacterium *P. aeruginosa* QDIO-6 (8.0 μg/mL), though with less activity compared to the antibiotic chloramphenicol (2.0 μg/mL) [61]. Similarly, *ent*-aspergoterpenin C (**12**) and 7-O-methylhydroxysydonic acid (**13**) were also reported to inhibit *P. aeruginosa* with MIC values of 8.0 and 32.0 μg/mL respectively. However, the monoterpenoid compounds (**1**) and (**2**) showed no antibacterial action against the pathogen [53]. In the same vein, other newly isolated monoterpenoids, aspergerthinacids A and B (**3**–**4**), were reportedly inactive against *S. aureus* [54]. While compounds (**133**) and (**134**) did not exhibit strong activity (MIC > 32 μg/mL), (5*S*,6*S*)-16,17-dihydroophiobolin H (**135**) exhibited inhibitory effects against *P. aeruginosa* at a concentration of 8.0 μg/mL. Notably, the *β*-H at C-6 is critical for the stronger activity of ophiobolin (**135**) compared to (**134**) [88]. Isolate (**59**) obtained from the ethyl acetate extract of *A. puniceus* A2 weakly inhibited *S. aureus* ATCC 29213 [73]. Millmerranones A−F (**211–216**) displayed no growth inhibition against *S. aureus* ATCC 25923, with half inhibitory concentration (IC_50_) > 30 μM [108]. Compound (**17**) did not exhibit activity against *S. aureus* ATCC 25923 at the highest tested concentration of 200.0 μg/mL compared to the control methicillin (MIC, 0.5 μg/mL) [63]. Antibacterial activities of compound (**15**) against the pathogenic strains of *S. aureus* (ATCC 29213), *K. pneumonia* (ATCC 13883), *E. faecalis* (ATCC 29212), *A. baumannii* (ATCC 19606), and methicillin-resistant *S. aureus* have also been reported [60]. Drimanenoids C–D (**45**–**46**) and F (**48**) showed inhibition against methicillin-resistant *S. aureus*, with inhibition diameters of 2.66 ± 0.47, 4.66 ± 0.47, and 5.00 ± 0.00 mm respectively [70]. The aperterpenes (**193–194**) had no inhibitory activity against *S. aureus* and *P. aeruginosa* at a concentration > 32 μg/mL [102]. 6-deoxyaspergiloid C (**109**) exhibited no activity against the growth of *S. aureus* (MIC, > 100 μg/mL) [84]. The aculeatupenes (**100**) and (**101**) showed antibacterial effects against *S. aureus* ATCC 25923 and *P. aeruginosa* ATCC 27853, with MIC of 128 μg/mL [81].

## Strategies to maximize the structural architect of the new terpenoids against ESKAPE pathogens

*Aspergillus* species, undoubtedly, remain sources of terpenoids with intriguing, structural carbon frameworks. It has been stressed that terpenoids possess potential as substitutes in overcoming AMR [110, 111]. These unique new secondary metabolites have not been fully maximized in the quest for new therapeutic agents against ESKAPE pathogens. A critical analysis of the obtained data revealed that only a few of the newly isolated terpenoids have been screened or channeled for antibacterial activities against these pathogens. While some displayed no activity, a number of them showed appreciable antibacterial effects against the tested pathogens. There are many ways of maximizing and/or optimizing the potential therapeutic values of these vast arrays of unique compounds. One approach is to utilize combinatory therapy of these terpenoids with antibiotics. Combinatory therapy is a new weapon to fight against multidrug resistance [112]. It does not only foster the optimization of antibacterial efficacy, but also counters development of resistance. It has been shown as an effective means to increase the potency of treatment against multidrug-resistant bacteria. A study reported that natural terpene *S*-limonene decreased the MIC from 16 to 0.237 µg/mL when combined with antibiotic, rifampicin [113]. Diterpenoids salvipisone and aethiopinone reportedly enhanced the activity of the antibiotic oxacillin against* S*. *aureus*. They reduced *S*. *aureus* biofilm by 40% in combination with oxacillin [114].

Chemical derivatization or synthesis of analogues of these terpenoids with unusual carbon framework is another innovative strategy. Some common structural optimization strategies include (i) altering existing functional groups or introducing new ones to improve interactions with biological targets, (ii) changing the core structure of the terpenoids to explore different biological activities, (iii) modifying the spatial arrangement of atoms to enhance selectivity and potency, and (iv) conjugating a sugar unit to improve solubility and transport across membranes. In the case of fumigatanol (**5**), modifying hydroxyl groups to esters (–COOCH₃) or ethers (–OCH₃) could improve membrane permeability. Also, by expanding the 6-membered ring in Malfilanol C (**59**) to a 7-membered ring could improve its binding affinity with the biological targets. In drug development and modern rational medicinal chemistry, this approach does not only save costs, but also reduces the time in developing terpene-based therapeutics for these bacteria [115, 116]. Undertaking detailed mechanistic studies on the new terpenoids that showed antibacterial actions will drive the development of target therapeutic intervention. It is one thing is for these terpenoids to show bactericidal activity, and another to know how they exert the bactericidal actions. While there are many enzyme targets/mechanisms attributed to the current clinical antibiotics/drugs, it will be great to explore uncharted paths in antibiotic-pathogen enzyme targets [117–119]. Moreover, new drugs/antibiotics are more prone to bacterial resistance when they have targets/mode of action similar to those currently being managed to treat infections. This pivotal approach offers one advantage to combat multidrug resistant infections caused by ESKAPE pathogens. Another ‘unpopular’ alternative intervention that might be profitable is to ‘repurpose’ other terpenoids that have not been studied for their anti-ESKAPE pathogen activities. The bulk of these new terpenoids as revealed from the available data has been driven by anticancer/cytotoxic research. Thus, massive antibacterial screening of this chemical library of novel terpenoids against ESKAPE members might result in lead/hit identification, optimization, and development.

## Concluding remarks and future outlook

The rise in antimicrobial resistance (AMR) from ESKAPE pathogens coupled with the need for new and effective drugs has necessitated developing and designing new antimicrobial drugs and/or alternative strategies for curbing these pathogens. Recent investigations on the natural products from the *Aspergillus* species offer the possibility of finding promising lead drug candidates. In this paper, we systematically synthesized and holistically documented newly isolated terpenoids from this genus (covering 2019–April 2024). We also examined their therapeutic prospects in the fight against ESKAPE members. The main *Aspergillus* species involved in the production of these terpenoids include the “culprits” *A. terreus*, *A. sydowii*, *A. versicolor*, *A. fumigatus*, *A. nidulans*, and *A. flavus* among others. In total, 217 new terpenoids were isolated. Among which, 81 are sesquiterpenoids, 60 are meroterpenoids, and 44 are sesterterpenoids. Pharmacologically, a few of these terpenoids exhibited appreciable in vitro antibacterial effects against ESKAPE pathogens. However, in vivo efficacy testing as well as the mechanism of their antibacterial drug targets is necessary for comprehensive evaluation of their drug-like potency. Importantly, a larger percentage of the compounds have not been fully characterized for their antibacterial activity. Thus, there is a need for these compounds to be subjected to in vitro and in vivo testing for their activity against ESKAPE pathogens and others that are being added to the WHO list of pathogens of concern. Additionally, innovative strategies, including chemical derivatization and combination therapy should be employed to optimize the bioactivity of compounds with antimicrobial activity, particularly those that have new biological targets providing in-depth understanding of their mechanisms of action, and opening new opportunities for antimicrobial development in the future.

## Data Availability

All the data in the manuscript are obtained from included references and available upon request.

## Abbreviations

AMR: Antimicrobial resistance
ATCC: American type culture collection
DMAPP: Dimethylallyl pyrophosphate
DMOA: 3,5-Dimethylorsellinic acid
ESKAPE: ***E****nterococcus faecium*, ***S****taphylococcus aureus*, ***K****lebsiella pneumoniae*, ***A****cinetobacter baumannii*, ***P****seudomonas aeruginosa*, and ***E****nterobacter* species
FPP: Farnesyl diphosphate (FPP
GGPP: Geranyl-geranyl- diphosphate
IPP: Isopentenyl pyrophosphate
MDR: Multidrug resistance
MEP: 2-C-methylerythritol phosphate
MIC: Minimum inhibitory concentration
MVA: Mevalonic acid
MRSA: Methicillin-resistant *S. aureus*
NPs: Natural products
PDR: Pandrug-resistance
WHO: World Health Organization
XDR: Extensive drug resistance
